# Seeking Meaningful Innovation: Lessons Learned Developing, Evaluating, and Implementing the Electronic Patient-Reported Outcome Tool

**DOI:** 10.2196/17987

**Published:** 2020-07-29

**Authors:** Carolyn Steele Gray

**Affiliations:** 1 Bridgepoint Collaboratory for Research and Innovation Lunenfeld-Tanenbaum Research Institute Sinai Health System Toronto, ON Canada; 2 Institute of Health Policy, Management and Evaluation Dalla Lana School of Public Health University of Toronto Toronto, ON Canada

**Keywords:** digital health, implementation, meaningfulness

## Abstract

Digital health solutions, in particular information communication technologies, often experience implementation failures leading to slower adoption than expected. This implementation challenge has spurred the development of frameworks to help navigate this uncertain and messy process. These frameworks point to environmental, organizational, individual, and technological factors that can drive or hinder implementation, with some in the field suggesting that perceived value may play a pivotal role. However, the concept of value can have varying meanings and be challenging to operationalize as a means to support implementation. Attending to philosophical and psychological meaningfulness for users and organizations in which technologies are adopted may offer a useful lens, by linking perceived value to individual behavior changes often required as part of implementing digital health technologies. Lessons learned from developing, evaluating, and implementing the electronic Patient-Reported Outcome (ePRO) tool demonstrate how qualitative methods can be used to uncover meaningfulness. By drawing from this example and other similar studies, this viewpoint offers suggestions on how future inquiry could deepen an understanding of meaningful innovation to help drive the implementation of digital health technologies.

## The Implementation Challenge Facing Digital Health

While digital technologies have transformed many industries worldwide, health systems remain laggards in adopting innovative and disruptive technology. In a recent reflection, Jadad and Jadad Garcia lament the glacial crawl of innovation adoption in health systems, stating that “sadly, the promises of information and communication technologies to transform healthcare services remain unfulfilled” [1]. Global health leaders have recognized this gap and are establishing strategies to support wider adoption of technologies that are viewed as foundational for robust health systems [2,3]. Included in these strategies is attention to overcoming implementation challenges that have slowed adoption. Implementation factors such as adaptability of the innovation, the implementation climate, the policy and system environment, and user characteristics are key hindrances [4,5]. In her viewpoint paper in the 20th anniversary issue of the *Journal of Medical Internet Research*, Buis argues that to achieve the vision of digital health, the next decade of inquiry requires greater attention to these implementation challenges [6].

Several theoretical frameworks and models of implementation have emerged and been applied to digital health. Two promising models include the Value-Proposition Design (VPD) [7], and the Non-adoption, Abandonment, Scale-up, Spread, and Sustainability (NASSS) framework [8]. These frameworks represent an important step forward in implementation science, offering applied and practical approaches that build on heuristic models like the Consolidated Framework for Implementation Research (CFIR) [9]. VPD and NASSS include attention to multiple implementation factors, and both suggest that the value proposition of a given technology is a critical driver of its implementation, an idea also reflected within the World Health Organization guidelines on adopting digital health solutions [10]. The importance of the perceived and evidenced value of these technologies has spurred calls for better evaluation standards of digital health products to drive adoption [11].

Although perceived value is one of several factors to consider, the above frameworks suggest it to be a central concern as it can *drive behavior change* in the individuals and organizations needed to implement a technology. However, the concept of value can be understood in many ways and through multiple disciplinary lenses. This viewpoint suggests *meaningfulness* as one potentially useful approach to uncover how perceived value can drive implementation.

## What is the Meaning of Meaning?

The notion of “meaning” in thinking of technology adoption in healthcare is not a new idea. The concept of “meaningful use” of electronic health records (EHRs) emerged in 2010 in an attempt to establish rules for how EHRs are to be used in order to improve quality and efficiency [12]. While this functionally focused notion of meaning has an important place, it may be too narrow to encompass different interpretations of meaning held by users. A broader understanding is offered through philosophical approaches exploring how individuals perceive that they are living meaningful lives. Some philosophers have argued that we see our lives and actions as being meaningful when we feel we are promoting good in the world, experiencing subjective satisfaction, and/or achieving our aims and life goals [13]. While the intention of this commentary is not to dive into the deep and rich philosophical literature on meaning, this approach to meaning can offer a lens to help explore and perhaps overcome some of the challenges experienced when implementing technology in health care.

Perceived meaningfulness is related to the implementation of digital health solutions in two ways. First, meaningfulness drives behavior, and behavior change is needed when adopting new technology. Social and cognitive psychology theories have shown how group interactional processes and individual cognitive and emotional processes motivate behavior change needed when implementing new practices [14], like when adopting a new electronic medical record or including telemonitoring as part of clinical care. Social determination theory, which suggests individuals’ goals and aspirations (one of the components of meaningfulness mentioned earlier), are key drivers of behavior [15]. Social determination theory has also been used to understand patient adherence to digital health interventions [16].

For providers, psychological meaningfulness associated with work has been shown to incent better engagement with that work. As Kahn [17] describes in his foundational paper on the subject, psychological meaningfulness in work is “*the harnessing of organization members’ selves to their work roles; in engagement, people employ and express themselves physically, cognitively, and emotionally during role performances.*”

Here Kahn connects meaning in work to a broader sense of self, which can, in turn, act as a powerful motivator to drive behavior. Classic motivation theorist Maslow viewed meaningfulness as one of several higher-order needs which humans strive for through the ongoing process of self-actualization [18]. Meaningfulness in work is argued to help individuals reach a level of self-actualization where individuals are driven by the work itself, rather than completing a task. Building on Maslow’s theory, Chalofsky and Krishna suggest meaning *in* work occurs when work speaks to an individual’s whole self and is perceived to be *good work*. When perceived meaning *in* work is paired with meaning *at* work, where individuals feel emotionally committed to their organizational environment, they will be subsequently more engaged and willing to go “above and beyond” [19]. This feeling of *meaningfulness* in work represents a “deeper level of intrinsic motivation” [19]. Tapping into that level of motivation to change can be crucial in technology implementation, where resistance to change is a significant barrier to implementing digital health solutions [4].

Second, while individuals’ sense of meaning in their lives can drive their behavior, it can also be influenced by the technologies they use. Technology itself represents an artifact in the social and work environment that can influence meaningfulness to individuals. There may be a risk in assuming the technology is simply a tool to manipulate, having little to do with what is important to people and their identities. Another perspective, grounded in a sociological approach, suggests technology plays an important role in how we understand ourselves, our work, and our environment. As Lupton so eloquently argues, health technologies are “*sociocultural products located within pre-established circuits of discourse and meaning. [Technologies] are active participants that shape human bodies and selves as part of heterogeneous networks, creating new practices and knowledge*” [20].

As such, when seeking to drive implementation of technology, we not only need to determine what value this brings to the individuals interacting with the technology as suggested by VPD and NASSS, but we also need to unpack how using the technology may change perceptions of value and meaning over time.

**Figure 1:**
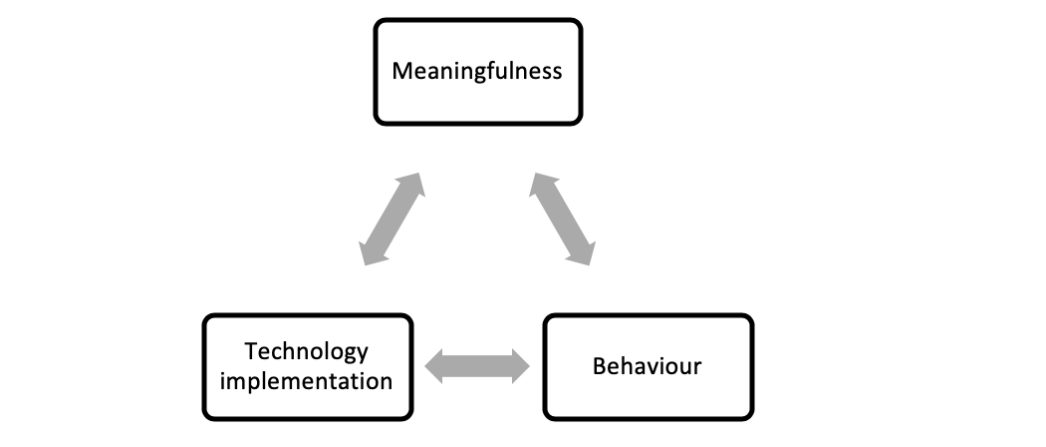
Meaningfulness in digital health.

Figure 1 offers a simple visualization of this proposed interrelationship between meaningfulness, behavior, and technology implementation.

## Seeking Meaningful Innovation

If meaningfulness is a key driver to implementation, then it is essential to uncover what is meaningful to users of systems within their organizations and system contexts. VPD and NASSS offer a set of questions to guide the implementation process iteratively. These questions are not only useful in uncovering objective opportunities and challenges in implementation (eg, whether there are necessary resources and regulations needed to adopt a particular technology) but can be used to uncover meaningfulness as well (eg, how individuals perceive a shared problem to be solved).

In adopting this approach, however, two challenges can arise. First, those asking the questions may struggle to pick up on meaningfulness because users will have different abilities and willingness to express what is meaningful. Simply put, you may ask the right question, but the answers may not be articulated in a way that meaningfulness can be easily understood. Second, it can be challenging to attend to what is important and meaningful to the many different users of a system which can include providers, administrators, patients, and families involved in health service delivery; all of whom have different professional and personal backgrounds, aims and goals, and personal perceptions of meaning.

Drawing on multiple *qualitative methods* can help not only ask the right questions at the right times but unpack and interpret the answers with an ear for meaningfulness. To demonstrate how this approach could be put into practice, the remaining sections draw on experiences implementing the electronic Patient-Reported Outcome (ePRO) tool, a mobile app and web-based platform designed to support goal-oriented care in primary care settings. Qualitative methods used to develop, evaluate, and implement ePRO across multiple settings offer lessons on how what was meaningful to patients, providers, and their organizations influenced the adoption of technology. Experiences with this technology are compared to other literature emphasizing meaningfulness to drive the implementation of digital health technologies.

## Developing for Meaningfulness

A focus on meaningfulness suggests a shift from merely seeing technology as a tool to support a process toward a deeper understanding of a user’s experience of receiving or delivering care in a particular context. User-centered co-design is a popular approach to employ the experiences and views of users to guide development [21]. It is essential to recognize that the process requires analytic work and iterative dialogue to link articulated desires for functionality, to why that function is important to users.

In the first rounds of developing the ePRO tool [22-24], interpretive description was used to understand user experiences. This method allows for situated and contextualized analysis with a view toward practical application of findings [25], which supported an iterative building of the interpretation of meaningfulness and drove the development of the technology. After undergoing multiple rounds of co-design, usability analysis found that although the technology had many shortcomings, it was highly used by patients, because they perceived it as meaningful [24].

The ARCHIE framework similarly suggests that the development of telehealth and telecare products must be “anchored in what matters to users” through iterative co-creation driven by a phenomenological lens [26]. The framework aligns with philosophical and sociological traditions that emphasize meaningfulness to users, in this case, patients, suggesting the use of phenomenological and ethnographic approaches to uncover meaningfulness and guide development.

## Evaluating for Meaningfulness

As the development of the ePRO tool moved from design into the iterative evaluation of usability testing and pilot trial, the focus on meaningfulness to both patient and provider users became even more important. This phase required the next step of linking meaningfulness to other components of the intervention beyond just the technology, looking at contexts, processes, and mechanisms that drive any change in patient outcomes. A narrative analysis of patient and provider interview data was used to identify dominant themes in the stories they told of using the technology [27].

Critical to both patient and provider stories was how the technology linked to what was important in their lives and work. For patients, this meant it supported their care goals (achieving personal aims) and reinforced a sense of shared accountability to those aims with their provider. Providers recounted that they were willing to put in more time and energy to learn and use the new technology, adapting workflows and processes, when the technology was perceived as important to their patients (linked to a sense of doing *good work*). When they viewed the technology as meaningful, patients and providers changed their behavior to support the regular use of the tool.

Other evaluation methods that have been applied to similar complex technology interventions have viewed meaningfulness as a central component. For example, Gomersall and colleagues suggest adopting network-based evaluation approaches, like social networking or realist approaches, to pick up on social and individual values and meaningfulness in evaluations of ambient assistive living technologies [28].

## Using Meaningfulness to Drive Implementation

The ePRO example and other studies indicate that meaningfulness may play an important role in the implementation of technologies. In these examples, meaningfulness is linked to personally held values and beliefs of individuals interacting with technologies, demonstrating that when technology aligns with those beliefs, it may be more likely to be used. However, meaningfulness is only one factor among other implementation drivers occurring at organizational and system levels. Uncovering meaningfulness for organizations and systems may also be required to support implementation. One approach is to consider how a given technology aligns to the *why,* or the vision, of an organization or care model. The critical point is to not just attend to that vision but also think about the guiding principles and values that drive organizations toward their goal. The disconnect between technology and what providers perceive as being foundational to their work and organization is a significant barrier to implementation [29].

Ideally, technologies would fit the aims and activities of the model of care as well as the guiding principles and values of the organizations and individuals delivering and seeking care. The ePRO tool was co-designed with those engaging in goal-oriented, person-centered care models. When spreading the tool to new environments as part of the pragmatic trial (see protocol [30]), it was most successful in primary care clinics that also had a strong vision of person-centered care delivery along with processes they could adapt to meet that aim. Although we are still analyzing findings from this study, observationally we found that where there was alignment, the ePRO tool was more likely to be perceived as valuable to providers and patients leading to changes in how patients engaged in their care, and how providers managed patients and communicated with each other around supporting patient goals. Preliminary analysis of trial data suggests alignment between technology and the organization’s vision and guiding principles can play an important role in implementation. A deep dive into the ethnographic data planned for this year will provide additional insight into this relationship.

## Future Work

The experience of developing, evaluating, and implementing the ePRO tool, and the emerging literature in this space suggests that meaningfulness matters. In particular, philosophical and psychological meaningfulness may be playing an important role in the process of implementation. The ideas presented are based on just a few examples and require more empirical testing. While meaningfulness to users was explored as part of the ePRO study, the importance of meaning as related to implementation emerged throughout the study; as such we did not start explicitly looking for connections between meaningfulness and other implementation constructs until we got to later stages and had an opportunity to reflect on what was learned.

This viewpoint is intended to start a conversation about the role of meaningfulness in implementation. Many questions are yet to be explored, including: is there a unique understanding of meaningfulness that pertains to technology? How does meaningfulness interact with other components of implementation? Can strong empirical evidence of the value of a technology overrule intrinsic perceptions of meaning held by users? How does meaningfulness relate to other socially grounded concepts like ethics and equity when implementing technologies?

Perhaps the more important takeaway from this viewpoint is that philosophical and psychological meaningfulness for those who engage with technologies in their work and lives plays a role that deserves attention. Adopting qualitative methods can serve to uncover meaningfulness for diverse users and organizations, and could help to drive implementation decisions. Future work can help to uncover how we can adapt and build research methods to place meaningfulness at the center of our implementation efforts so that perhaps our reflections on the state of digital health a decade from now are much less grim than those we see today.

## Abbreviations

CFIR: Consolidated Framework for Implementation Research
EHR: electronic health record
ePRO: electronic patient-reported outcome
NASSS: Non-adoption, Abandonment, Scale-up, Spread, and Sustainability
VPD: Value-Proposition Design
